# Expression of Platelet-Derived Growth Factor Alpha and Beta Receptors in Primary Tumor Cells of Patients with Renal Cell Carcinoma

**DOI:** 10.5152/tud.2025.24154

**Published:** 2025-05-21

**Authors:** Maria Volkova, Dmitry Khochenkov, Anna Olshanskaya, Yulia Khochenkova, Elyso Solomko, Saida Ashuba, Ilya Tsimafeyeu

**Affiliations:** 1 FSBI N.N. Blokhin National Medical Research Center for Oncology, Moscow, Russian Federation; 2 Togliatti State University, Togliatti, Russian Federation; 3 Bureau for Cancer Research - BUCARE, New York, USA

**Keywords:** Platelet-derived growth factor, renal cell carcinoma, survival

## Abstract

**Objective:**

**:** This study aimed to assess the expression of platelet-derived growth factor receptors alpha and beta (PDGFRα/β) in primary tumor cells of patients with renal cell carcinoma (RCC).

**Methods:**

Platelet-derived growth factor receptors alpha and beta expression was analyzed in RCC specimens from 65 RCC patients (pT1a-T4N_any_M_any_) using immunohistochemistry. Expression levels were quantified using the semi-quantitative H-score (HS) method, and correlations between PDGFRα/β expression and tumor characteristics were evaluated. The impact of PDGFRα/β expression on patient survival was also examined.

**Results:**

Platelet-derived growth factor receptor alpha was expressed in the cytoplasm and membrane of 58.5% of primary RCC cells, with an HS of 62.9 ± 8.4, significantly higher than PDGFRβ expression (44.6%; 26.6 ± 5.3; *P* > .05). Platelet-derived growth factor receptor alpha expression correlated with tumor grade (r = 0.471; *P* < .0001) and the pN+ category (r = 0.280; *P* = .024). Platelet-derived growth factor receptor beta expression correlated with tumor grade (r = 0.286; *P* = .021), venous tumor thrombosis (r = 0.263; *P* = .034), M+ category (r = 0.305; *P* = .014), and adrenal metastases (r = 0.306; *P* = .041). Neither PDGFRα nor PDGFRβ expression levels influenced patient survival.

**Conclusion:**

Platelet-derived growth factor receptor alpha was more highly expressed in RCC cells compared to PDGFRβ. Overexpression of PDGFRα/β was associated with higher tumor grade and advanced RCC stages, though it did not affect patient survival.

**Main Points**

- Primary tumor cells of renal cell carcinoma patients express platelet-derived growth factor receptor alpha and, to a lesser extent, platelet-derived growth factor receptor beta.
- Overexpression of platelet-derived growth factor receptors correlated with high tumor grade (G3-4) of renal cell carcinoma and tumor burden, including venous thrombosis (platelet-derived growth factor receptor beta), nodal involvement (platelet-derived growth factor receptor alpha), distant metastases (platelet-derived growth factor receptor beta), and adrenal metastases (platelet-derived growth factor receptor beta).
- Any significant influence of platelet-derived growth factor receptor alpha or platelet-derived growth factor receptor beta expression on patient prognosis was not found.

## Introduction

Renal cell carcinoma (RCC) is a significant global health concern, with its pathogenesis influenced by multiple genetic and molecular factors. In metastatic cases, treatment strategies have evolved to target key molecular pathways involved in tumor progression. Modern therapies focus on blocking growth factors and receptors with tyrosine kinase activity, which plays a crucial role in tumor cell proliferation and survival. These targeted approaches have improved patient outcomes, offering more effective and personalized treatment options.

Platelet-derived growth factor (PDGF) target genes are regulated by the hypoxia-inducible factor (HIF) signaling pathway, which is frequently overexpressed in RCC. Platelet-derived growth factor plays a crucial role in tumor progression through several mechanisms: autocrine stimulation of cancer cells, promotion and maintenance of angiogenesis, and regulation of tumor interstitial fluid pressure.^1^ The expression of PDGF and its receptors (PDGFRs) has been observed across various human cancers.^2^ However, few studies have specifically explored the role of this signaling pathway in RCC. The aim of this study was to investigate the expression of PDGFRα and PDGFRβ in RCC cells and assess their impact on tumor growth characteristics and patient prognosis.

## Materials and Methods

### Patients

Formalin-fixed paraffin-embedded (FFPE) tissue samples were obtained from 65 RCC patients following nephrectomy, prior to any systemic therapy, between December 2018 and January 2020. Eligible patients were 18 years or older and had not received any treatment prior to surgery. Final inclusion in the study was confirmed after the histological diagnosis of RCC.

Follow-up evaluations were conducted every 3 months in patients with non-metastatic disease and every 2 months in patients with metastatic disease using computed tomography until confirmed disease progression, the date of death, or the last visit if the patient was still alive.

This prospective observational study was approved by the Ethics Committee of FSBI N.N. Blokhin National Medical Research Center for Oncology, Moscow, Russian Federation (Approval no.: 08012019; Date: 08.01.2019) and was conducted in accordance with Good Clinical Practice guidelines, the Declaration of Helsinki, and applicable local regulations. All patients provided written informed consent before any study-related procedures.

### Immunohistochemistry

Surgically obtained tumor tissue samples were prospectively collected and analyzed. Routine morphological examinations were performed on all samples. Platelet-derived growth factor receptor alpha and PDGFRβ expression in RCC tissues were evaluated using immunohistochemistry, with antibodies obtained from Abcam and Santa Cruz Biotech via the Invitrogen immunohistochemical staining kit. Expression levels were assessed using a semi-quantitative method, measuring staining intensity (0, 1+, 2+, and 3+) and calculating the percentage of stained cells (0%-100%). The immunohistochemical expression level, represented as the H-score (HS), was calculated by multiplying the percentage of stained cells by their staining intensity.^3^

### Statistical Analysis

The primary objective was to evaluate PDGFRα and PDGFRβ expression in RCC. Secondary objectives of the study included the correlation of PDGFRα and PDGFRβ expression levels with tumor characteristics such as grade, stage, presence of venous invasion, and metastatic burden. Additionally, the prognostic significance of these biomarkers in terms of survival was evaluated.

Data analysis was conducted using SPSS Statistics 19 (IBM SPSS Corp.; Armonk, NY, USA). The Pearson correlation coefficient (r) was used to assess correlations between variables, and its significance was evaluated. Receiver Operating Characteristic curves were generated to evaluate the predictive accuracy of variables, with the most prognostically significant cut-off values determined from the curve coordinates. Overall survival (OS) was calculated from the date of surgery to the last follow-up or death in the intent-to-treat (ITT) population. Disease-free survival (DFS) was measured from the date of radical surgery to the date of recurrence, and progression-free survival (PFS) from the date of cytoreductive surgery to the date of RCC progression. Survival rates were assessed using the Kaplan–Meier method, with differences determined by log-rank tests. Univariate and multivariate Cox regression analyses were used to identify significant prognostic factors for survival.

## Results

### Patient Characteristics

The study included 65 patients with RCC at stages pT1a-T4N_any_M_any_. Patient characteristics were described in Table 1. The median age was 59.0 years (range 33-79), with a male-to-female ratio of 1.9:1. All patients were diagnosed with RCC, with the majority having unilateral kidney tumors (59 patients, 90.8%) and 6 patients (9.2%) presenting with bilateral tumors. The median size of the largest renal tumor was 10 cm (range 2.5-26 cm). Tumor venous invasion was observed in 50 patients (76.9%). At the time of surgery, 45 patients (69.2%) had distant metastases, with 22 (33.8%) showing solitary metastases and 23 (35.4%) having multiple metastatic lesions. Additionally, 11 patients (16.9%) had metastases in more than one location. The most common sites of metastasis were the adrenal gland (28 cases, 43.1%), lungs (22 cases, 33.8%), bones (5 cases, 7.7%), and liver (2 cases, 3.1%).

All patients underwent nephrectomy with extended retroperitoneal lymph node dissection, and thrombectomy was performed in 50 patients (76.9%). In 28 patients (43.1%), additional tumors beyond the primary tumor were removed, including adrenalectomy in 24 patients (36.9%), contralateral partial nephrectomy in 1 patient (1.5%), lung resection in 1 patient (1.5%), and removal of bone metastasis in 1 patient (1.5%). Forty patients (61.5%) underwent radical surgery, while 25 patients (38.5%) had cytoreductive surgery.

Histological examination confirmed RCC in all primary tumor samples, with the clear cell subtype being the most common (59 cases, 90.8%). Non-clear cell RCC was identified in 6 cases (9.2%). Tumor grade was G1-2 in 29 patients (44.6%) and G3-4 in 36 patients (55.4%). Twelve patients (18.5%) were at stage pT1-T2, while 53 patients (81.5%) were at stage pT3-T4. Perinephric fat invasion was found in 29 cases (44.6%). Thrombotic masses removed during thrombectomy were consistent with kidney tumors, and venous wall invasion was observed in 4 cases (6.2%). Distant retroperitoneal lymph node metastases were present in 12 patients (18.5%), with more than one lymph node affected in 8 patients (12.3%). Histological analysis confirmed that tumors removed from other sites were RCC metastases originating from the primary tumor in the kidney.

Patients who underwent radical surgery were followed up. Among the 25 patients who had cytoreductive surgery, 22 (88.0%) received systemic therapy. This included cytokine therapy for 3 patients with pulmonary metastases and targeted anti-angiogenic therapy for 19 patients.

### Expression Levels and Outcomes

Platelet-derived growth factor receptor alpha expression (62.9 ± 8.4 HS) was observed in the cytoplasm and on the membrane of 58.5% of primary renal carcinoma cells, while PDGFRβ expression was lower, found in 44.6% of samples (26.6 ± 5.3 HS), though this difference was not statistically significant (*P* > .05).

The relationship between PDGFRα/β expression levels and various tumor characteristics—including tumor laterality, size, histological subtype, grade, pT stage, perinephric fat invasion, tumor-associated venous thrombosis, venous wall invasion, pN and M categories, and the presence of metastases in lymph nodes, lungs, bones, adrenal glands, and liver—was examined. Overexpression of PDGFRα and PDGFRβ was significantly associated with unfavorable tumor characteristics and higher tumor burden. Specifically, PDGFRα overexpression correlated with higher tumor grade and positive nodal status (pN+), while PDGFRβ overexpression was linked to the extent of tumor-associated thrombosis, the presence of distant metastases, and adrenal metastases (*P* < .05 for all correlations). Significant correlations are presented in Table 2. The median follow-up time was 19.9 months (range 1-133 months). The median OS and cancer-specific survival for the 65 patients were 43.8 months and 52.1 months, respectively. Among the 40 patients who underwent radical surgery, the median DFS was 79.2 months, while the median PFS for the 25 patients who underwent cytoreductive nephrectomy was 7.4 months. No significant impact of PDGFRα or PDGFRβ expression on OS, DFS, or PFS was observed (all *P* > .05).

## Discussion

The most common and well-studied RCC subtype is clear cell renal cell carcinoma,^4^ which is associated with a high rate of inactivating mutations of the von Hippel-Lindau gene leading to overexpression of HIF^5^^,6^ and its targets, primarily growth factors and receptor tyrosine kinases.^7^ ^8^ Platelet-derived growth factor/platelet-derived growth factor receptor is one of the key HIF-dependent signaling pathways.^9^ The PDGF family proteins transduce signals inside the cell by binding to PDGF tyrosine kinase receptors α and β. The PDGF/PDGFR signaling pathway is an extensively studied mechanism of attracting perivascular smooth muscle cells and pericytes, which underlies the pro-angiogenic activity of PDGF.^10^ An experimental glioma model showed that PDGF enhanced the proliferation of both tumor and endothelial cells.^11^ Data on the expression and prognostic role of PDGFRα and PDGFRβ in kidney cancer are scarce and require further research.

This study was carried out on prospectively collected surgical samples of RCC patients. Immunohistochemistry with a semi-quantitative assessment of protein production by tumor cells, using both the number of stained cells and the staining intensity (HS), was selected as a tool to evaluate expression. This method is highly reproducible, has proven its value in early works,^3^ and is currently widely used by other researchers.^12^^-^ ^14^

We found PDGFRα and PDGFRβ expression in half of the patients. Similar results were reported in early published papers on the subject. Sulzbacher et al^15^ (2003) conducted an immunohistochemical study of surgical RCC tissue samples from 112 patients and revealed PDGFRα expression in 87.5% of the cases. In a series of 1423 RCC tissue specimens studied by Song et al^16^ (2014), cytoplasmic overexpression of PDGFRβ was detected in 32.8% of clear cell renal carcinomas. Cumpănas et al^17^ (2016) found PDGFRβ expression in one-third of 50 RCC specimens, while the expression levels were low in all stained tissue samples.

We also observed a significant correlation of PDGFRα and PDGFRβ overexpression with high tumor grades (G3-4) and significant tumor burden: the extent of tumor thrombosis (only PDGFRβ), pN+ (only PDGFRα), and M+ (only PDGFRβ) stages, including metastases to the adrenal glands (PDGFRβ). Similar results were obtained by Sulzbacher et al^15^ PDGFRα overexpression (staining of >38.8% of 500 cells) correlated with the tumor grade (G3-4). In another work that included tissue samples from 314 RCC patients, the authors found a direct correlation of perivascular PDGFRβ overexpression with the tumor’s advanced stage and grades G3-4.^18^ In contrast, Tawfik et al^19^ (2007), who studied surgical specimens from 62 radical surgery patients with RCC, showed that PDGFRα expression did not correlate with the tumor characteristics. Notably, recent findings align with those of a previously published study that had a shorter follow-up period.^20^

Regarding survival outcomes, no significant impact of PDGFRα or PDGFRβ expression on OS, DFS, or PFS was observed. However, Tawfik et al^19^ evaluated the prognostic value of a number of molecular markers in 62 radical surgery patients with RCC and revealed that PDGFRα expression was an independent risk factor for OS and bone metastases. Frödin et al^18^ (2017), who studied tissue samples of 314 RCC patients, demonstrated that PDGFRβ overexpression was associated with a significant decrease in OS. Univariate analysis reported by Sulzbacher et al^15^ showed an association between PDGFRα overexpression and a decrease in DFS in 112 patients with RCC; however, it lost its significance in a multivariate analysis. Platelet-derived growth factor receptor alpha overexpression was found to be an independent risk factor for PFS in a study by Kusuda et al^21^ (2013). One limitation of this study is the relatively small sample size, which may affect the generalizability of the findings and results in each cohort. Additionally, the observational design and the lack of functional studies limit the ability to establish a direct causal relationship between PDGFR expression and tumor progression in RCC. Moreover, this cohort had a high proportion of patients with both thrombi and metastases, which may be attributed to the study being conducted in specialized centers with expertise in kidney cancer treatment and the surgical removal of tumor thrombi.

In conclusion, the expression of the tyrosine kinase receptors PDGFRα and, to a somewhat lesser extent, PDGFRβ, was detected on the surface and in the cytoplasm of primary tumor cells of patients with RCC stage pT1a-T4N0/+M0/+. Overexpression of the studied markers was found to be significantly correlated with high tumor grade (G3-4) (PDGFRα,β) and significant tumor burden: the extent of tumor venous thrombosis (PDGFRβ), pN+ stage (PDGFRα), and M+ stage (PDGFRβ), as well as adrenal metastases (PDGFRβ). There was no influence of PDGFRα and PDGFRβ expression levels on the prognosis of RCC patients.

## Figures and Tables

**Table 1.** Patient Characteristics

Baseline Characteristics	Patients (n = 65)	
	Number	%
+**Age, years, median (range)**	59.0 (33-79)	
+**Gender**		
Male	43	66.2
Female	22	33.8
+**Side of kidney lesion**		
Right	35	53.8
Left	24	36.9
Both	6	9.2
+**Size of primary tumor, cm, median (rage)**	10.0 (2.5-26.0)	
+**Stage рТ**		
рТ1а	2	3.1
рТ1b	2	3.1
pT2a	8	12.3
рТ3а	10	15.4
рТ3b	29	44.6
рТ3c	12	18.5
pT4	2	3.1
+**Tumor thrombus**	50	76.9
+**Stage рN1**	12	18.5
+**Stage М1**	45	69.2
+**Number of metastatic lesions**		
1	22	33.8
>1	23	35.4
+**Number of organs with metastases**		
1	34	52.3
>1	11	16.9
+**Metastatic sites**		
adrenal	28	43.1
lung	22	33.8
bone	5	7.7
liver	2	3.1
+**Histological type**		
Clear-cell	59	90.8
Papillary, type 1	2	3.1
Papillary, type 2	3	4.6
Chromophobe	1	1.5
+**Histopathologic grading **		
G1	3	4.6
G2	26	40.0
G3	27	41.5
G4	9	13.8
+**History of nephrectomy**		
Radical	40	61.5
Cytoreductive	25	39.5
+**Other surgeries**		
Partial nephrectomy for contralateral kidney tumor	1	1.5
Adrenalectomy	24	36.9
Lung resection	1	1.5
Liver resection	1	1.5
Bone resection	1	1.5
Systemic therapy for patients with disease progression (n = 25)	22	88.0
Cytokines	3	12.0
Targeted therapy	19	76.0

**Table 2.** Correlation of Platelet-Derived Growth Factor Receptor Alpha and Platelet-Derived Growth Factor Receptor Beta Expression Levels in Kidney Tumor Cells with Tumor Characteristics in Renal Cell Carcinoma Patients

	Pearson Correlation (r), Two-Tailed Significance	PDGFRα	PDGFRβ
Tumor grade (G)	R	0.471**	0.286*
	Sig (two-tailed)	0.000	0.021
Tumor venous thrombosis level	R	0.114	0.263*
	Sig (two-tailed)	0.367	0.034
рN+ stage	R	0.280*	0.132
	Sig (two-tailed)	0.024	0.296
M+ stage	R	−0.040	0.305*
	Sig (two-tailed)	0.749	0.014
Adrenal metastases	R	−0.273	0.306*
	Sig (two-tailed)	0.070	0.041

PDGFRα, platelet-derived growth factor receptor alpha; PDGFRβ, platelet-derived growth factor receptor beta.

*Correlation is significant at 0.05 (two-tailed).

**Correlation is significant at 0.01 (two-tailed).

## Data Availability

The data that support the findings of this study are available upon request from the corresponding author.
